# Announcing the 2016 *Pharmaceuticals* Travel Award for Young Investigators

**DOI:** 10.3390/ph9030035

**Published:** 2016-06-27

**Authors:** Jean Jacques Vanden Eynde

**Affiliations:** Editor-in-Chief, MDPI AG, Klybeckstrasse 64, Basel CH-4057, Switzerland; jean-jacques.vandeneynde@ex.umons.ac.be

For the first time in its short history, our journal is able, this year, to support a young researcher in the field of medicinal chemistry by offering a travel grant of 800 CHF. That sum will help the laureate to attend an international conference and to share his/her scientific results by presenting an oral communication or a poster. We have been delightfully surprised to receive as many as 83 outstanding applications for that grant, which confirms the expanding interest in our activities and the growing number of visitors to our website. On the other hand, selecting only one candidate among these excellent investigators emerged as an uneasy and frustrating task for the selection committee. Unfortunately, our limited budget does not allow us to offer more than one award, which we deeply regret.

In order to guarantee the impartiality of the selection process, the 83 applications have been evaluated by two independent groups in our editorial office and two short lists of approximately 20 individuals were established. Amazingly, seven identical names appeared in each list. The corresponding files of those seven candidates were then sent to a panel of the Evaluation Committee comprised of *Pharmaceuticals* editorial board members. By adding the scores attributed to each applicant by this Committee, we are pleased to announce that the winner is Dr. Zesergio Melo Jerez, of Colombia, who obtained his PhD degree in 2014 from the Universidad Nacional Autónoma de México, Mexico City, Mexico. On behalf of the Evaluation Committee, the editorial staff and all our readers, we sincerely congratulate him.


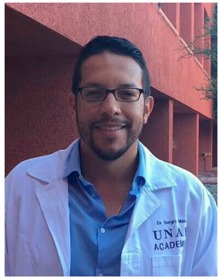


Dr. Zesergio Melo Jerez received a degree as a bacteriologist from the Universidad Industrial de Santander, Colombia in 2002 and a PhD in biochemical sciences from the Universidad Nacional Autónoma de México. During his PhD-student days, he worked on the understanding of the physiology of renal potassium-chloride cotransporters. He is currently working as a postdoctoral fellow in the Institute of Neurobiology at the Universidad Nacional Autónoma de México, Querétaro, Mexico and his application was warmly supported by Dr. Gonzalo Martínez de la Escalera. His work focuses on the role of vasoinhibins in the nervous system. He will present an oral communication at the 39th annual meeting of the Japan Neuroscience Society to be held from 20–22 July, in Yokohama, Japan. His presentation is entitled “Vasoinhibins Block NGF-Induced Neurite Outgrowth and Decrease Survival of PC12 Cells”.

Once again we congratulate the laureate and we thank all applicants and their mentors for the admirable selection of high quality abstracts we received. We hope that the remarkable breakthroughs described therein will be accomplished. 

